# KI and WU Polyomaviruses in Children, France

**DOI:** 10.3201/eid1403.071206

**Published:** 2008-03

**Authors:** Vincent Foulongne, Natalie Brieu, Eric Jeziorski, Amandine Chatain, Michel Rodière, Michel Segondy

**Affiliations:** * Montpellier University Hospital, Montpellier, France

**Keywords:** KI human polyomavirus, WU human polyomavirus, respiratory tract disease, children, France, letter

## Abstract

KI and WU Polyomaviruses in Children, France

**To the Editor:** Two new members of the *Polyomaviridae* family, provisionally named *Karolinska Institutet virus* (KIPyV) and *Washington University virus* (WUPyV), have been recently discovered (*1*,*2*). These new polyomaviruses were identified by screening human respiratory secretions with molecular tools. KIPyV and WUPyV are genetically related to the BK virus and the JC virus, the 2 known members of the family *Polyomaviridae* that affect humans.

In France, from November 2006 through June 2007, nasopharyngeal aspirates were obtained from 537 children who were <5 years of age and who had acute respiratory tract disease. The aspirates were tested for respiratory syncytial virus (RSV); influenza virus types A and B; parainfluenza virus types 1, 2, and 3; and adenoviruses (AdVs) by direct immunofluorescence assay. The aspirates were also tested for human metapneumovirus (HMPV) by an enzyme immunoassay (HMPV EIA, Biotrin, Lyon, France) and for the human bocavirus (HBoV) by PCR (*3*). Samples were placed on MRC5 cell monolayers for virus isolation.

Nucleic acid extracts were tested for KIPyV and WUPyV DNA by PCR. KIPyV detection was performed by using a nested PCR approach that targeted the VP1 capsid gene as described by Allander et al. (*1*). For WUPyV detection, primers targeted the predicted 3′ end of the large T antigen coding region as described by Gaynor et al. (*2*). The amplification specificity was assessed by sequencing the PCR product; sequences were deposited in GenBank (WUPyV isolates, accession no. AM778536–48; KIPyV isolates, accession no. AM849808–10).

At least 1 type of virus was identified for 271 (50.5%) children. The viruses found were RSVs in 175 (32.6%), HBoVs in 54 (10.0%), HMPVs in 50 (9.3%), rhinoviruses/enteroviruses in 11 (2%), influenza A viruses in 8 (1.5%), human AdVs in 6 (1.1%), and parainfluenza type 3 viruses in 4 (0.7%) samples. Aspirates were not tested for coronaviruses; detection of rhinoviruses/enteroviruses was likely low because cell culture is less sensitive than molecular assays.

A total of 13 (2.4%) samples were positive for WUPyV; of these 4 (30.8%) were co-infected with another virus. The 13 children with samples positive for WUPyV had a median age of 11.2 (2–48) months and the male/female sex ratio was 2.2. KIPyV DNA was detected in samples from 3 (0.6%) boys (ages 10, 18 and 30 months); 1 of those samples was co-infected with RSV and HMPV.

Sequences of WUPyV and KIPyV isolates varied little from each other and from other GenBank sequences, which suggests that these polyomaviruses are genetically conserved viruses. Clinical characteristics of children infected with WUPyV and KIPyV are retrospectively recorded (Table). All children recovered and were able to return home within 1 to 10 days, with the exception of 1 child. This child had been hospitalized since birth for congenital myopathy; nosocomial acquisition or vertical transmission of the WUPyV is suspected.

**Table:** Clinical findings for 13 children infected with WU polyomavirus and 3 children infected with KI polyomavirus*

Sex/age, mo	Copathogen	Fever, °C	CRP, mg/L	SaO_2_, %	WBC, x 10^3^ cells/µL	Chest radiograph findings	Diagnosis	Clinical signs and symptoms
WU polyomavirus								
M/2	–	37.9	8.3	NA	9.4	Hyperinflation	Pneumonia	Cough, dyspnea
M/12	–	39.2	112.0	NA	18.2	Hyperinflation, interstitial infiltrate	Bronchiolitis	Cough, dyspnea
F/20	–	38.7	56.0	NA	13.7	Hyperinflation	Atypical pneumonia	Cough, respiratory distress, diarrhea
F/12	–	37.8	15.0	96	12.3	Hyperinflation	Bronchiolitis	Cough, dyspnea, pharyngitis
M/10	–	39.0	90.3	NA	18.0	Normal	Idiopathic fever	Fever
F/14	–	38.9	20.8	95	11.0	Hyperinflation, interstitial infiltrate	Atypical pneumonia	Cough, wheezing, dyspnea
F/10	RSV	38.0	14.0	NA	17.2	Hyperinflation, interstitial infiltrate	Bronchiolitis	Cough, respiratory distress, dyspnea
M/6	RSV	38.0	NA	89	NA	Hyperinflation	Bronchiolitis	Respiratory distress, wheezing
M/48	HMPV	36.8	15.0	94	13.0	Hyperinflation	Asthma	Cough, respiratory distress
M/24	–	37.0	23.0	98	13.9	Hyperinflation	Asthma	Cough, respiratory distress
M/2	–	38.5	38.5	70	16.0	Normal	Idiopathic fever	Cough, fever
M/11	HBoV	39.0	5.5	94	6.2	Interstitial infiltrate	Atypical pneumonia	Cough, fever
M/10	–	37.0	10.0	97	18.0	Alveolar syndrome	Congenital myopathy	Respiratory distress
KI polyomavirus								
M/10	RSV, HMPV	39.0	NA	NA	NA	Hyperinflation	Bronchiolitis	Cough, wheezing, otitis, diarrhea
M/30	–	39.2	<5.0	NA	11.0	Interstitial infiltrate	Influenza-like pneumonia	Cough, dyspnea, otitis, sore throat
M/18	–	38.9	23.0	96	14.8	Hyperinflation, atelectasis	Asthma	Cough, dyspnea, wheezing

*CRP, C-reactive protein; SaO_2_, oxygen saturation; WBC, white blood cells; NA, not available; HMPV, human metapneumovirus; RSV, respiratory syncytial virus; HBoV, human bocavirus.

Our data are in agreement with the 2 original reports that show that the new KI and WU polyomaviruses may be detected in respiratory secretions from patients with respiratory diseases (*1*,*2*). WUPyV was detected in 2.4% of children <5 years of age who were hospitalized with respiratory tract disease, which is in accordance with the 2% incidence reported by Gaynor et al. (*2*). The 0.6% prevalence observed for KIPyV PCR is also in agreement with data reported from Sweden (*1*) and Australia (*4*). A seasonal change in the presence of WUPyV was not observed; however, all KIPyV isolates were found only during January.

KIPyV and WUPyV were mainly detected in samples from children with lower respiratory tract disease, such as bronchiolitis or atypical pneumonia, and in samples from children with exacerbated asthma. These preliminary data on the likely role of these viruses as respiratory pathogens need to be interpreted with caution. Aspirates were obtained only from those with observed symptoms; no asymptomatic controls were tested. Detection of WUPyV and KIPyV in respiratory samples may simply reflect a respiratory transmission route as previously suggested for BK virus and JC virus (*5*). Another virus was in aspirates from 31% of the children with KI and WU polyomaviruses. Substantial rates of codetection were also reported in the initial descriptions of both WUPyV and KIPyV (*1*,*2*). More recently, Bialasiewicz et al. reported a 25% rate of codetection of KIPyV with another pathogen (*4*). These high rates of co-infection raise questions about the real pathogenic role of these viruses.

As with other polyomaviruses, WUPyV and KIPyV could establish persistent and latent infections with likely asymptomatic reactivations (*5*), and detection of these viruses could also reflect a long-term shedding from previous acute episode. Recently published studies have not shown a pathogenic role for these new polyomaviruses in respiratory tract disease (*6*,*7*); however, more comprehensive studies are needed to elucidate whether both KIPyV and WUPyV have any clinical relevance.

## References

[1] Allander T, Andreasson K, Gupta S, Bjerkner A, Bogdanovic G, Persson MAA, Identification of a third polyomavirus. J Virol. 2007;81:4130–6. 10.1128/JVI.00028-07 17287263 PMC1866148

[2] Gaynor AM, Nissen MD, Whiley DM, Mackay IA, Lambert SB, Wu G, Identification of a novel polyomavirus from patients with acute respiratory tract infections. PloS Pathog. 2007;3:e64. 10.1371/journal.ppat.0030064 17480120 PMC1864993

[3] Foulongne V, Olejnik Y, Elaertz S, Perez V, Rodière M, Segondy M. Human bocavirus in French children. Emerg Infect Dis. 2006;12:1251–3. 16965707 10.3201/eid1208.060213 PMC3291226

[4] Bialasiewicz S, Whiley DM, Lambert SB, Wang D, Nissen MD, Sloots TP. A newly reported human polyomavirus, KI virus, is present in the respiratory tract of Australian children. J Clin Virol. 2007;40:15–8. 10.1016/j.jcv.2007.07.001 17706457 PMC7172449

[5] Randhawa P, Vats A, Shapiro R. The pathobiology of polyomavirus infection in man. Adv Exp Med Biol. 2006;577:148–59. 16626033 10.1007/0-387-32957-9_10

[6] Abed Y, Wang D, Boivin G. WU polyomavirus in children, Canada. Emerg Infect Dis 2007;13:1939–41. 10.3201/eid1312.070909 PMC2876769 18258053

[7] Norja P, Ubillos I, Templeton K, Simmonds P. No evidence for an association between infections with WU and KI polyomaviruses and respiratory disease. J Clin Virol. 2007;40:307–11. 10.1016/j.jcv.2007.09.008 PMC7172997 17997354

